# Omphalolith: Une présentation déroutante

**DOI:** 10.4314/pamj.v8i1.71152

**Published:** 2011-04-01

**Authors:** Mariem Bounouar, Asmae Hatimi, Mariame Meziane, Ouafae Mikou, Fatimazahra Mernissi, Amal Bennani, Sanae Chehbouni, Taoufik Harmouch, Afaf Amarti

**Affiliations:** 1 Service de Dermatologie, Centre Hospitalier Universitaire Hassan II, Fès, Maroc; 2 Service d’anatomie pathologique, Centre Hospitalier Universitaire Hassan II, Fès, Maroc

**Keywords:** Omphalolithe, ombilic, tumeur

## Abstract

Les omphalolithes sont des concrétions crayeuses constituées de débris de kératine et de sébum, qui s’accumulent au niveau de l’ombilic. Peu d’observations ont été rapportées dans la littérature. Elles sont l’apanage des ombilics profonds, et sont parfois associées à une mauvaise hygiène. Elles peuvent passer inaperçues pendant des années ne se révélant qu’en cas d’inflammation, d’infection ou d’ulcération. Nous rapportons un cas particulier d’omphalolithe révélé par une tumeur ombilicale d’aspect framboisé.

## Introduction

Les omphalolithes sont l’apanage des ombilics profonds. Ils peuvent passer inaperçus pendant des années ne se révélant qu’en cas d’inflammation, d’infection ou d’ulcération. Nous rapportons un cas particulier d’omphalolithe révélé par une tumeur ombilicale.

## Patient et observation

**Figure 1:**
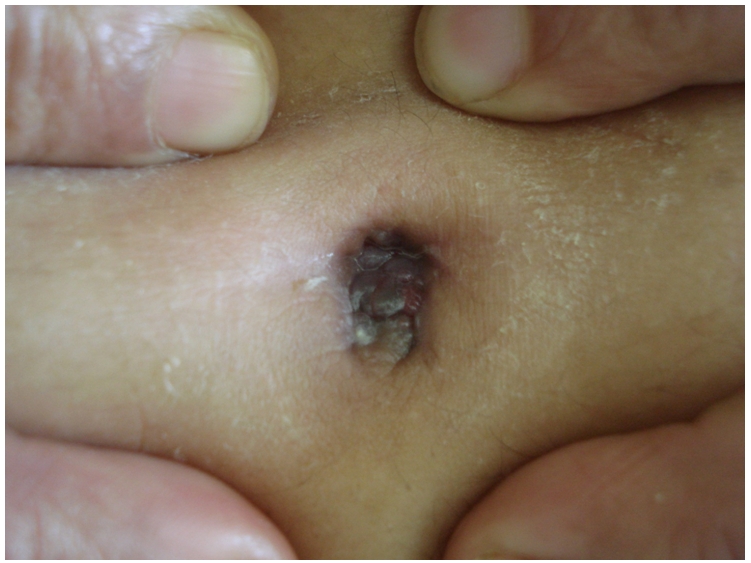
Tumeur ombilicale d’aspect framboisé, polylobée, à surface mamelonnée, de 2.5 cm de grand axe

Mme R.N âgée de 35 ans était admise pour bilan étiologique d’une tumeur ombilicale indolore évoluant depuis un an. La patiente avait rapporté la notion d’écoulement ombilical séreux depuis un an sans autres signes locaux ni généraux associés. L’examen clinique avait retrouvé une tumeur ombilicale d’aspect framboisé, polylobée, à surface mamelonnée, de 2.5 cm de grand axe (Figure 1).

**Figure 2:**
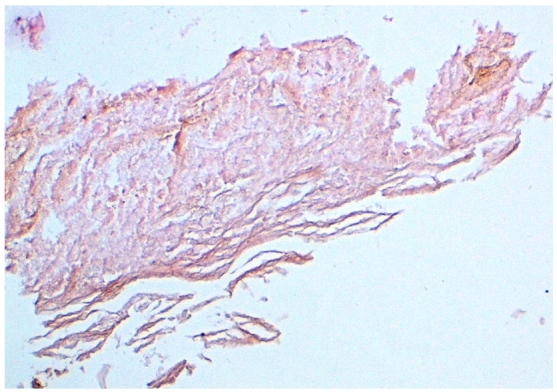
Coupe histologique montrant des lamelles de kératine feuilletées et aérées, avec une substance amorphe sans structure tissulaire (Coloration Hématoxyline Eosine; grossissement×50)

L’étude histologique d’une biopsie cutanée avait objectivé un granulome inflammatoire non spécifique. L’étude bactériologique du liquide ombilical était négative. L’échographie abdominale a montré une infiltration du tissu sous-cutané sous-jacent, et le fistulo-scanner n’a pas objectivé de trajet fistuleux. Un curetage complet de la tumeur a été réalisé; des débris blanchâtres profondément incrustés dans l’ombilic ont été détachés. L’étude histologique de ces débris a objectivé des lamelles de kératine feuilletées et aérées, avec une substance amorphe sans structure tissulaire (Figure 2). Nous avons donc conclu au diagnostic d’une omphalolithe compliquée d’un granulome inflammatoire.

## Discussion

Le diagnostic différentiel d’une tumeur ombilicale comprend plusieurs maladies dont les anomalies de développement embryonnaire, les métastases ombilicales (Nodule de Sœur Mary-Joseph), le mélanome et l’endométriose. Chez notre patiente, l’aspect de la tumeur et la présence d’écoulement nous ont initialement induits en erreur. Une anamnèse plus approfondie nous avait révélé la notion de manipulation de l’ombilic par la patiente, et l’extériorisation de débris noirâtres par moments.

Les omphalolithes ou omphalokératoliths [1] sont des concrétions crayeuses constituées de débris de kératine et de sébum qui s’accumulent au niveau de l’ombilic [2]. L’oxydation des lipides ainsi que l’accumulation de mélanine peuvent être responsables de la coloration noire de certains omphalolithes [1]; Dans d’autres cas, l’omphalolithe peut garder un aspect blanchâtre, notamment en l’absence de contact avec l’air, dans ce cas elle peut être révélée par un granulome pyogénique [3].

Les omphalolithes sont en général l’apanage du sujet âgé et des ombilics profonds, et sont souvent associées à une mauvaise hygiène [4]. Elles peuvent passer inaperçues pendant des années ne se révélant qu’en cas d’inflammation, d’infection ou d’ulcération [2]. Elles sont fréquentes dans la population japonaise âgée, à cause d’une certaine superstition selon laquelle, le fait de toucher au « sésame ombilical » pourrait déclencher des douleurs abdominales [4].

Le nettoyage prudent à la curette mousse assure aussi bien le diagnostic que le traitement des omphalolithes. L’histologie permet d’éliminer les autres diagnostics différentiels, notamment un mélanome en cas de tumeur noire.

## Conclusion

Nous voulons à travers cette observation rappeler cette pathologie ombilicale bénigne qui peut prêter confusion avec d’autres lésions dont le pronostic est réservé.

## Conflits d’intérêts

Les auteurs déclarent n’avoir aucuns conflits d’intérêts

## Contribution des auteurs

Tous les auteurs ont contribué à la prise en charge diagnostique et thérapeutique de la patiente et à la rédaction de ce cas clinique.
